# Innovations in Thoracic Oncology and the Promise of Liquid Biopsies with Dr. Luis Raez

**DOI:** 10.3390/cancers16040799

**Published:** 2024-02-15

**Authors:** Viviana Cortiana, Alexandra Van de Kieft, Harshal Chorya, Jade Gambill, Chandler H. Park, Yan Leyfman

**Affiliations:** 1 Department of Medical and Surgical Sciences (DIMEC), University of Bologna, 40126 Bologna, Italy; 2 Cornell University, Ithaca, NY 14853, USA; amv85@cornell.edu; 3 Medical College Baroda, Vadodara 390018, India; harshalchorya548@gmail.com; 4 Parker University, Dallas, TX 75229, USA; jade.l.gambill@outlook.com; 5 Norton Cancer Institute, Louisville, KY 40202, USA; 6 Icahn School of Medicine at Mount Sinai, New York, NY 10029, USA

**Keywords:** thoracic oncology, lung cancer, liquid biopsies, biomarkers

## Abstract

**Simple Summary:**

This article examines the significance of liquid biopsies in the field of thoracic oncology, particularly regarding the diagnosis and treatment of malignancies. These blood-based biopsies, which utilize circulating tumor DNA (ctDNA), are presented as a less invasive method for both the diagnosis and monitoring of cancer cells and offer a dynamic means of tracking tumors, allowing for personalized treatment approaches and real-time information about the tumor’s status throughout its natural history. The analysis of biomarkers through blood samples could therefore lessen the need for more invasive tissue biopsies. The article also addresses the valuable role circulating biomarker analyses play in allowing treatment plans to be modified according to the genetic changes observed in the tumor. The importance of adapting to genetic aberrations that may develop during treatment is highlighted, emphasizing the need for timely adjustments to ensure a therapy’s effectiveness. In summary, the article underscores the potential of liquid biopsies to revolutionize thoracic cancer care by offering a minimally invasive and personalized approach to diagnosis and treatment, emphasizing the importance of their further advancement.

**Abstract:**

Thoracic oncology continues to pose a great threat to human health as one of the most common forms of cancer. Liquid biopsies present a transformative approach to treating patients affected by these types of diseases by providing a less invasive genetic overview of the tumor, aiding in both diagnostic and treatment measures. The primary objective of this article is to examine the prospects of liquid biopsies in managing thoracic malignancies and to present barriers to their usage as demonstrated by Dr. Luis Raez. In examining why molecular diagnostics continue to be employed together with more traditional methods, this article presents the next steps in the clinical application of blood-based cancer screening. Future cancer diagnosis and treatment aim to prioritize circulating biomarker analyses based on their potential for the detection and monitoring of thoracic cancers. Liquid biopsies are favored thanks to their reduced invasiveness with respect to traditional treatments. The further study of clinical biomarkers and technological advancements are thus pivotal to enhance the clinical applicability of this method. In conclusion, this blood-based analysis offers a promising route by which the diagnosis, treatments, and outcomes of thoracic cancer can be improved.

## 1. Introduction

**Figure 1:**
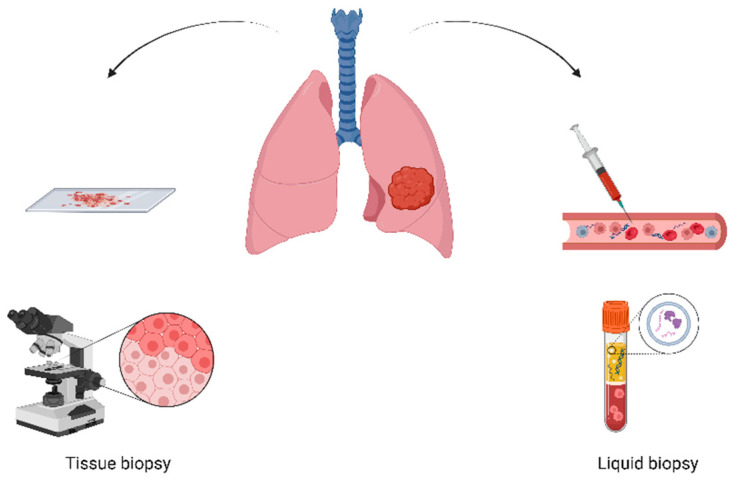
Graphic simplified representation of liquid and tissue biopsy methods in lung thoracic malignancies.

Thoracic oncology involves the diagnosis and treatment of malignancies primarily within the chest, including the esophagus and lungs, which are the most common forms of thoracic tumors [1]. Lung cancer, constituting a predominant form of thoracic tumors, stands as a leading cause of cancer-related mortality globally. Despite significant strides in the field, the 5-year survival rate for thoracic cancers, especially non-small cell lung cancer (NSCLC), remains alarmingly low, emphasizing the urgency for innovative treatment protocols and enhanced diagnostic methods [1]. Early-stage diagnosis significantly improves survival rates, underscoring the imperative need for novel diagnostic approaches. Liquid biopsies emerge as a promising, less-invasive method for detecting and monitoring cancer cells circulating in the bloodstream. By using biomarkers to find circulating tumor DNA (ctDNA), liquid biopsies are a dynamic way to both diagnose tumors and track how well they are responding to treatment. Liquid biopsies present a less invasive method of detecting and monitoring cancer cells present within one’s blood. As with non-cancerous human cells, malignant cells undergo death and replacement and following their breakdown are released from the tumor and divulged into the bloodstream. Liquid biopsies can therefore detect the presence of circulating tumor DNA (ctDNA) and offer a method for both diagnosis and the observation of a tumor’s response to specific courses of treatment [2]. Liquid biopsies thus utilize the detection of biomarkers circulating within the blood of patients [3] (Figure 1).

## 2. Clinical and Investigational Practice: Implementing Liquid Biopsies for Tumor Tracking

**Figure 2:**
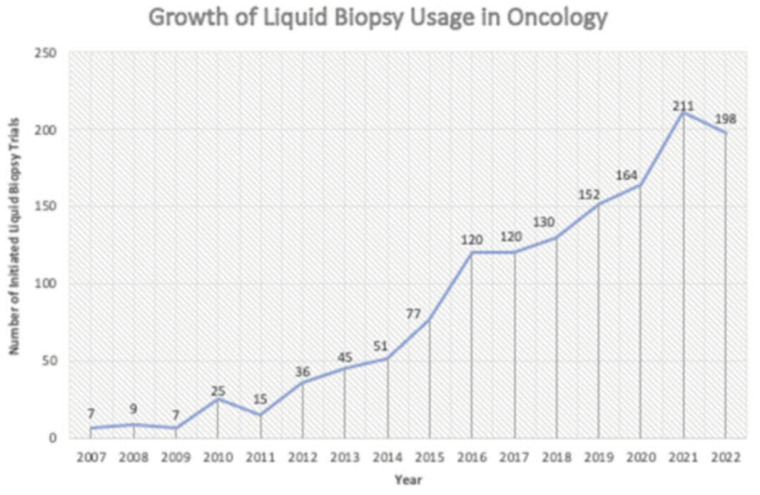
Number of Initiated Liquid biopsy trials in oncology, 2007–2022. [9].

Effective utilization of biomarkers requires integrating liquid biopsies into a rational, future-focused strategy for tracking tumors, and therefore it has recently been introduced and used in the context of clinical trials in oncology (Figure 2). A patient is typically diagnosed when there is a measurable increase in the amount of tumoral DNA in the blood. If the patient undergoes surgery and other treatments such as chemotherapy or immunotherapy and the amount of tumoral DNA decreases, the therapy may be later implemented if the patient presents recurrence and the tumoral DNA increases [4]. This scenario constitutes a more rational way to track tumors but is not usually implemented. The future of biomarkers entails finding a dynamic way to use these molecules to track the tumor at any specific time in their natural history. By using liquid biopsies, biomarkers can be tracked at every stage of the disease, offering an example of a method that ought to be used sooner rather than later [5]. Liquid biopsies also prove a vital role in identifying mutations that could implicate cancer, such as the T790M mutation which is present in about half of cancer patients [6]. The utility of liquid biopsies becomes evident when considering the challenges associated with repeated lung cancer biopsies. Conducting multiple tissue biopsies can be cumbersome, particularly given the inherent risks such as the potential for a small pneumothorax and the increased likelihood of complications in elderly patients with concomitant conditions like emphysema. Liquid biopsies offer a more accessible alternative, enabling the healthcare team to iteratively perform next-generation biomarker analysis without the need for invasive procedures, utilizing only blood samples [7,8]. In the context of advanced treatment using potent drugs, the emergence of resistant genetic aberrations is a common occurrence. Promptly identifying and adapting to these genetic changes is crucial for optimizing treatment effectiveness. Liquid biopsies provide a streamlined approach by facilitating repeated biomarker analysis and next-generation sequencing (NGS). This genetic analysis offers insights into the evolving nature of genes, allowing for swift adjustments in treatment strategies. By incorporating biomarker analysis and NGS, healthcare providers can efficiently monitor genetic alterations, thereby reducing the need for frequent tissue biopsies every time a patient experiences treatment failure.

## 3. Investigational Frontiers: Distinguishing PCR-Based and NGS-Based Analyses

Liquid biopsy has emerged as a powerful diagnostic tool for detecting tumor-specific gene mutations associated with a variety of cancers. This non-invasive technique can currently be employed to identify specific genetic alterations, including those in well-known cancer-related genes such as EGFR [10], KRAS [11], BRAF [12], TP53 [13], and PIK3CA [14]. Additionally, liquid biopsy allows for the assessment of Microsatellite Instability (MSI), a crucial factor in certain cancers like colorectal cancer and endometrial cancer [15]. The technique proves further valuable in detecting HER2 (Human Epidermal Growth Factor Receptor 2) amplification, a genetic alteration associated with specific types of breast cancer and gastric cancer [16] (Table 1).

Also, in the realm of thoracic oncology, Polymerase Chain Reaction (PCR) and Next-Generation Sequencing (NGS) studies serve as prominent tools for identifying cancer cells and deciphering the genetic alterations driving cancer growth [17,18]. PCR-based techniques exhibit exceptional efficacy in detecting minute quantities of circulating tumor DNA (ctDNA), facilitating the early identification of tumor presence. On the other hand, NGS-based analyses offer a broader genomic perspective, aiding in the identification of specific genetic mutations and alterations within the tumor.

Liquid biopsy can also identify ALK (Anaplastic Lymphoma Kinase) rearrangements in cancers such as non-small cell lung cancer (NSCLC), offering insights into treatment strategies [19]. Moreover, the detection of ROS1 gene rearrangements in lung cancer cases [20] and the identification of the EGFR T790M mutation. NGS offers therefore instrumental means by which NSCLC treatment resistance can not only be identified but isolated to a single mutation. The T790 mutation, for example, represents one of the most common resistance mutations observed frequently in NSCLC that can be efficiently recognized through NGS, allowing for treatment plans to be quickly altered [21]. The T790 mutation is found in the epidermal growth factor receptor gene (EGFR). Thus, when this mutation is pinpointed by NGS, the finding signals that future EGFR-targeted therapies may display reduced effectiveness, allowing a treatment team to alter provided therapies. With the potential to pinpoint a single mutation before clinical symptoms of resistance are manifest, cases can then be treated more efficiently as treatment quickly adjusts to the changing genetic landscape of a tumor. This technology eliminates a “one-size-fits-all” approach to thoracic oncology treatment and can tailor targeted treatment strategies to match the unique case of an individual.

While both PCR and NGS approaches possess their unique merits and drawbacks, NGS-based analysis is generally acknowledged for its heightened sensitivity and specificity. However, it is important to note that NGS-based analysis entails higher costs and demands more time compared to PCR-based analysis. Despite these considerations, both PCR and NGS analyses find applicability in thoracic oncology, with the choice of technology hinging on the individual needs of the patient and the clinical context. Navigating these unexplored territories allows for a careful selection of the most suitable analytical technique tailored to the clinical or investigative objectives at hand. Acquiring a nuanced understanding of the advantages and disadvantages inherent in PCR and NGS analyses enhances the precision of liquid biopsy applications in thoracic oncology, ultimately contributing to more informed decisions regarding personalized patient treatment and fostering advancements in scientific investigation.

**Table 1:** Genes and Gene Specific Mutations and Cancers that are commonly associated with these mutations.

Gene/Related Factors	Related Cancer
EGFR (Epidermal growth factor receptor)	Lung Cancer [10]
KRAS (Kristen Rat Sarcoma Viral Oncogene Homolog)	Pancreatic, Colon, Lung, Breast [11]
BRAF (B-Raf Proto-Oncogene)	Melanoma, Colorectal, Non-Small Cell Lung Cancers [12]
TP53 (Tumor Protein P53)	Ovarian, Esophageal, Colorectal, Head and Neck, Larynx, Lung [13]
PIK3CA (Phosphatidylinositol-4,5-Bisphosphate 3 Kinase Catalytic Subunit Alpha)	Colorectal, Breast, Colon, Endometrial, Brain, Skin, Ovarian, Gastric, Lung, Thyroid, Head and Neck, Cervical, Pancreatic, Esophageal, Liver/Biliary Tract, Pituitary, Urological, Leukemia/Lymphoma, Neuroblastoma [14]
MSI (Microsatellite Instability)	Adrenocortical Carcinoma, Cervical Cancer, Mesothelioma [15]
HER2 (Human Epidermal Growth Factor Receptor 2) Amplification	Breast, Gastric/Gastroesophageal, Ovary, Endometrium, Bladder, Lung, Colon, Head and Neck [16]
ALK (Anaplastic Lymphoma Kinase) Rearrangements	Neuroblastoma, NSCLC, Anaplastic Thyroid Cancer [19]
ROS 1 (ROS Proto-Oncogene 1, Receptor Tyrosine Kinase) Rearrangements	NSCLC [20]
EGFR T790M Mutation	NSCLC [21]

## 4. Integrating Liquid Biopsies and Overcoming Immunotherapy Challenges in Thoracic Oncology

Immunotherapy, a groundbreaking advancement in thoracic oncology, has exhibited substantial efficacy in treating lung cancer, particularly in stage four patients. Nevertheless, its effectiveness tends to reach a plateau after approximately a year, leading to progression in about half of the patients. Dr. Luis Raez’s research underscores that while immunotherapy benefits over 20% of lung cancer patients, the duration of its efficacy remains constrained [4]. The current imperative is to augment the success rate beyond the initial 12 months, especially for patients lacking specific genetic markers and variations. Regulatory cells may impede sustained immunotherapy effectiveness, resulting in transient improvements followed by a cessation in response. Ongoing research and clinical trials in the United States aim to overcome this challenge by developing products that target regulatory cells before continuing immunotherapy.

The integration of liquid biopsies into clinical and research practices in thoracic oncology represents a significant stride, necessitating a profound understanding of diverse treatment goals [7,8]. For individuals undergoing treatment with the intent of curing their cancer, liquid biopsies prove pivotal in assessing treatment efficacy and detecting minimal residual disease (MRD). This information empowers doctors to take a proactive approach in managing the disease. Conversely, for patients undergoing treatment to extend their lives, liquid biopsies serve to monitor disease progression and adjust treatment plans based on evolving genetic profiles. Specifically, next-generation sequencing (NGS) in liquid biopsies provides a comprehensive genetic analysis, aiding clinicians in selecting targeted therapies.

Recognizing the constraints of palliative care is crucial, given the evolving genetic heterogeneity that may pose challenges in identifying actionable targets. The incorporation of liquid biopsies should align with overarching treatment goals, considering ethical, logistical, and financial aspects. Additionally, the designs of clinical trials should be adapted to account for the unique opportunities and challenges presented by liquid biopsies in both curative and palliative settings. This approach ensures meaningful results for different types of thoracic oncology patients.

## 5. Conclusions

The future utility of liquid biopsies holds immense promise for enhancing both cancer diagnosis and the efficient monitoring of tumor progression. These analyses not only detect the presence of cancer but also track the sequence of DNA fragments released by the tumor, offering a dynamic insight into its genetic alterations [22]. As cancer cells undergo mutations or rearrangements, blood-based screenings can unveil these changes, enabling the care team to assess the need for treatment adjustments. The adaptable nature of cancer mutations suggests that tailored responses may be more effective, and blood-based biopsies facilitate a proactive and timely adjustment of treatment strategies. Compared to conventional tissue biopsies, liquid biopsies present compelling advantages. Beyond merely detecting thoracic cancer, they play a pivotal role in monitoring treatment responses and constructing a dynamic profile of the tumor’s evolution over time. Liquid biopsy assays efficiently identify mutations, contributing to the formulation of individualized treatment plans in a time-effective manner. The future trajectory of immunotherapy for treating thoracic cancers hinges on circumventing regulatory cells, which can impede the effectiveness of traditional treatments, allowing cancer to progress unimpeded. This lack of response may manifest from the outset of immunotherapy or later in the treatment course. The advent of circulating tumor DNA testing heralds a new era in thoracic oncology, wherein treatment becomes both personalized and minimally invasive.

In essence, liquid biopsies possess the potential to significantly elevate the standard of care for thoracic cancer patients, thereby improving health outcomes. The critical further development and advancement of liquid biopsy technologies are imperative for realizing this transformative potential.

## Data Availability

No patient data were directly utilized in this study.
